# Supplementary material for: Content-rich biological network constructed by mining PubMed abstracts
Source: BMC Bioinformatics. 2004 Oct 8;5:147. doi: 10.1186/1471-2105-5-147 (PMC528731; doi:10.1186/1471-2105-5-147)
Supplement: Additional File 2 — The original results of the above study (non-essential files are deleted to keep the file size under the limit set by BMC bioinformatics). [file 1471-2105-5-147-S2.bz2 › chilibotAdditionalFile2/dip20/762ID9732274E18149/html/RHLB_RNE.html]

 


 **RHLB** and **RNE** 
  
Found 14 abstracts in PubMed, retrieved 14.  
 

 What does Google say? 
 PDF only 
| .edu only 

---

**Interactive relationship** (e.g. stimulation, inhibition, etc)

**Neutral relationship**- Rather,  **Rne**  coordinates the activation of  **RhlB**  dependent on a 3 single stranded extension on RNA substrates.  Ref: 10521403 Genes Dev, 1999
- As expected, purified degradosomes, a multi protein complex containing, among others, RNase E  [ **RNE** ] , PNPase, and  **RhlB** , generate an authentic 147 residue RNase E  [ **RNE** ]  cleavage product from the rpsT mRNA in vitro.  Ref: 9642084 J Mol Biol, 1998
- **RhlB**  by itself has little activity but is strongly stimiulated by its interaction with RNase E  [ **RNE** ] .  Ref: 12035760 Biochem Soc Trans, 2002
- Earlier work has shown that the C terminal region of RNase E  [ **RNE** ]  is a scaffold for the binding of degradosome components and has identified specific RNase E  [ **RNE** ]  segments necessary for its interaction with polynucleotide phosphorylase PNPase,  **RhlB**  RNA helicase, and enolase.  Ref: 11134527 Proc Natl Acad Sci U S A, 2001
- DEAD box  **RhlB**  RNA helicase physically associates with exoribonuclease PNPase to degrade double stranded RNA independent of the degradosome assembling region of RNase E  [ **RNE** ] .  Ref: 12181321 J Biol Chem, 2002
- Our results demonstrate that the carboxy terminal half CTH of ribonuclease E  [ **RNE** ]  RNase E  [ **RNE** ]  contains the binding sites for the three other major degradosomal components, the DEAD box RNA helicase  **RhlB** , enolase, and polynucleotide phosphorylase PNPase.  Ref: 9732274 Genes Dev, 1998
- We also show that the C terminal half of E. coli RNase E  [ **RNE** ]  is both sufficient and necessary for its physical interaction with the 3 5 exoribonuclease polynucleotide phosphorylase, the  **RhlB**  helicase, and the glycolytic enzyme enolase, which are components of a degradosome complex.  Ref: 9751718 Proc Natl Acad Sci U S A, 1998
- RNase E  [ **RNE** ]  is a large multidomain protein with N terminal ribonucleolytic activity, an RNA binding domain and a C terminal scaffold that binds PNPase, enolase and  **RhlB** .  Ref: 12035760 Biochem Soc Trans, 2002
- Furthermore, polypeptides derived from the highly charged region of RNase E  [ **RNE** ] , containing the  **RhlB**  binding site, stimulate  **RhlB**  activity at least 15 fold, saturating at one polypeptide per  **RhlB**  molecule.  Ref: 9732274 Genes Dev, 1998

**Non-interactive relationship** (e.g. studied together, co-existance, homology, etc.)

- The most important recent developments have been the discovery that the degradosome a multiprotein complex containing an endoribonuclease RNase E  [ **RNE** ] , an exoribonuclease polynucleotide phosphorylase, and a DEAD box helicase  **RhlB**  has a central role in mRNA degradation.  Ref: 10684583 Bioessays, 2000
- The Escherichia coli degradosome is a multienzyme complex with four major protein components the endoribonuclease RNase E  [ **RNE** ] , the exoribonuclease PNPase, the RNA helicase  **RhlB**  and enolase.  Ref: 9383162 Mol Microbiol, 1997
- Our results demonstrate that a degradosome like complex containing RNase E  [ **RNE** ] , PNPase, and  **RhlB**  can form spontaneously in vitro in the absence of all other cellular components.  Ref: 10521403 Genes Dev, 1999
- Using an E. coli two hybrid system, together with BIAcore apparatus, we investigated the ability of three proteins, polynucleotide phosphorylase PNPase,  **RhlB**  RNA helicase, and enolase, a glycolytic protein, to interact physically and functionally independently of RNase E  [ **RNE** ] .  Ref: 12181321 J Biol Chem, 2002
- These results provide evidence for the first time that both functional and physical interactions of individual degradosome protein components can occur in the absence of RNase E  [ **RNE** ]  and raise the prospect that the RNase E  [ **RNE** ]  independent complexes of  **RhlB**  RNA helicase and PNPase, detected in vivo, may constitute mini machines that assist in the degradation of duplex RNA in structures physically distinct from multicomponent RNA degradosomes.  Ref: 12181321 J Biol Chem, 2002
- Furthermore, in the absence of RNase E  [ **RNE** ] , purified  **RhlB**  can carry out ATP dependent unwinding of double stranded RNA and consequently modulate degradation of double stranded RNA together with the exonuclease activity of PNPase.  Ref: 12181321 J Biol Chem, 2002
- Here, we show that, when PK is expressed in uninfected Escherichia coli cells, the C terminal half of RNase E  [ **RNE** ]  and the associated RNA helicase  **RhlB**  are heavily phosphorylated.  Ref: 11722741 Mol Microbiol, 2001
- These results suggest that MrsC, directly or indirectly, controls endonucleolytic processing of mRNAs that may be independent of the RNase E  [ **RNE** ]  PNPase  **RhlB**  multiprotein complex.  Ref: 9537393 J Bacteriol, 1998
- The relationship between the modification of RNase E  [ **RNE** ]  and  **RhlB**  and these mRNA stabilization effects, which may be relevant to the stability of late T7 mRNAs during infection, is discussed.  Ref: 11722741 Mol Microbiol, 2001
